# Pattern formation in a mean field model of electrocortical activity

**DOI:** 10.1186/1471-2202-14-S1-P149

**Published:** 2013-07-08

**Authors:** Lennaert van Veen, Kevin Green

**Affiliations:** 1 Faculty of Science, University of Ontario Institute of Technology, Oshawa, Ontario L1H 7K4, Canada

## 

Mean field models of cortical activity describe the electrical potentials and interactions of cortical neuron populations, coarse-grained over the scale of a few macro columns. Such models can be analysed as dynamical systems, in particular as time-dependent partial differential or integro-differential equations, which may have complicating aspects such as explicit time delays and stochastic terms. We analyse a model of intermediate complexity, formulated by Liley et al. [1]. This model describes an excitatory and an inhibitory population in a simple geometry, on which the effect of long-range connections is represented by a damped wave equations.

Although somewhat rudimentary from a physiological point of view, this model has been shown to predict several features of electrocortical dynamics rather well (see, e.g., [2,3] and refs therein) and is challenging to analyse mathematically. It consists of fourteen coupled partial differential equations with strong nonlinearities.

Where previous analysis on this model, and similar mean-field models, has used drastic simplifications, such as reduction to zero or one spatial dimensions or a single population, we developed tools for parsing the dynamics of the full-fledged equations [4]. Using the open-source library PETSc [5], we have implemented fully implicit time-stepping for the field equations and the tangent linear model, as well as arclength continuation for equilibrium and time-periodic solutions. All computations are performed in parallel using domain decomposition.

**Figure 1:**
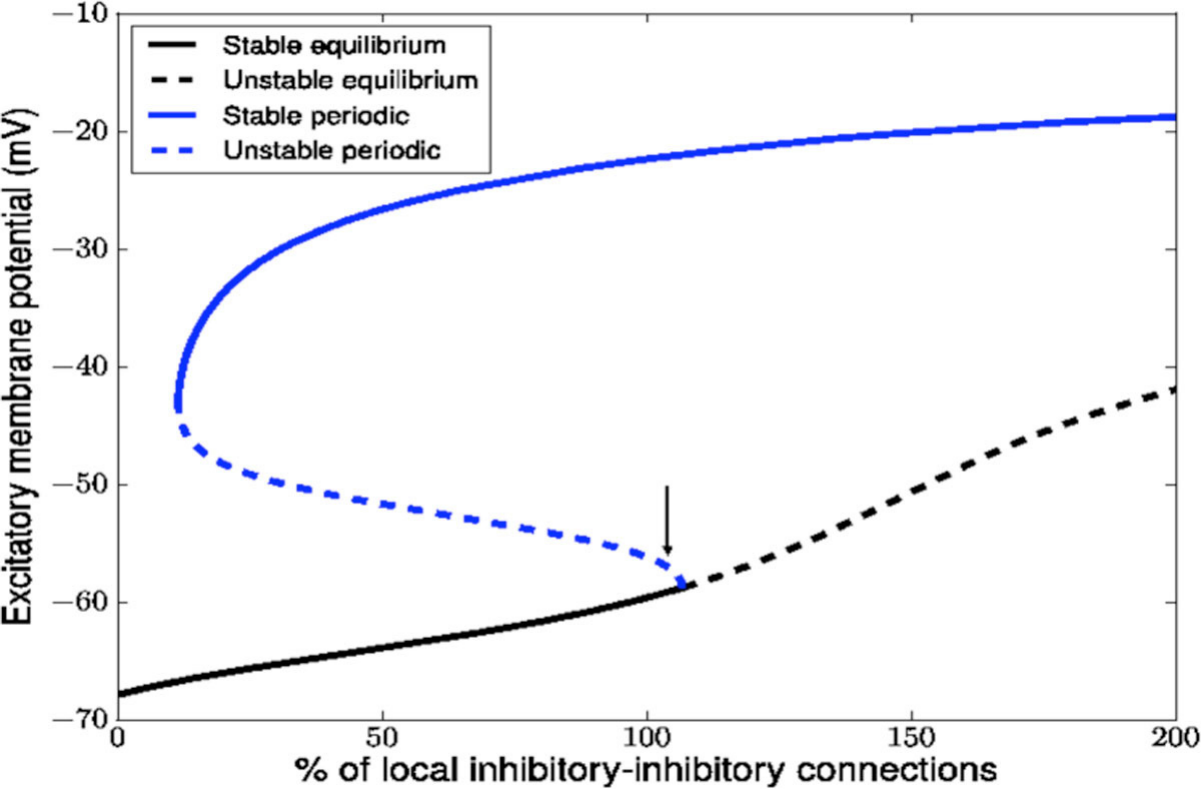
**Partial bifurcation diagram near the primary instability, which is a subcritical Hopf bifurcation**.

In the current application of these tools, we focus on physiologically interesting γ-range activity [3]. This activity is triggered by a Hopf bifurcation under small variations of the local inhibitory to inhibitory connection density. We computed the saddle-type periodic orbit that regulates the transient dynamics of perturbations to the base equilibrium state. Two snap shots of this orbit, computed on a 12.8 by 12.8 cm domain, with 0.5 mm resolution, are shown in Figure 1. The period of this orbit corresponds to a 12 Hz oscillation, whereas the final, attracting, state has a strong 40 Hz peak in the power spectrum [3].

In ongoing work we are investigating bifurcations of this periodic orbit, which can turn completely stable or give rise to more complicated solutions, such as quasi-periodic or chaotic. This dynamical systems approach to the analysis of mean-field models should give us more insight in the complex model behaviour.

**Figure 2:**
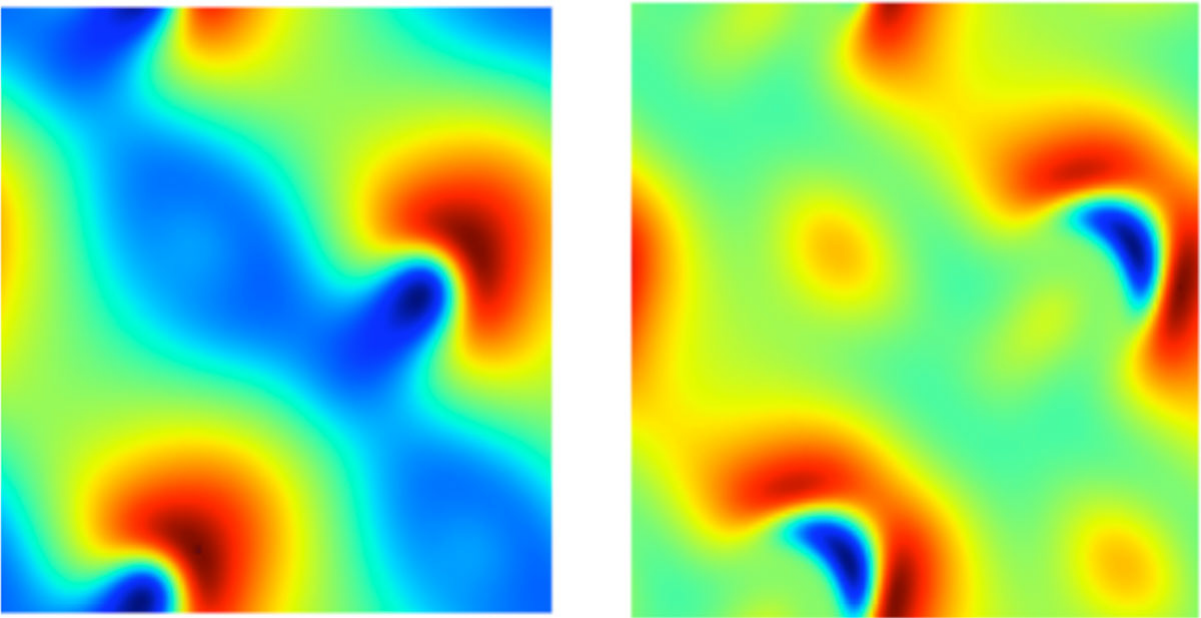
**Two snap shots of the excitatory membrane potential along the saddle periodic orbit**. The potential ranges from -67 mV (dark red) to -52 mV (dark blue).
